# 

**Published:** 1881-05

**Authors:** 


Vol. I. MAY, 1881. No. 5.

### THE HOMŒOPATHIC PHYSICIAN:

### A MONTHLY JOURNAL OF MEDICAL SCIENCE.

"*Magna est veritas et praevalebit.*"

E. J. LEE, M. D.

EDITOR.

